# Creep prediction of cellulose based materials by extrapolation of short term experiments

**DOI:** 10.1038/s41598-026-38132-3

**Published:** 2026-02-13

**Authors:** B. Emek Abali, Reza Afshar, Kristofer Gamstedt, Orlando Girlanda

**Affiliations:** 1 Division of Applied Mechanics, Uppsala University, Box 35, 751 03 Uppsala, Sweden; 2 Hitachi Energy Research Sweden, Forskargränd 7, 722 26 Västerås, Sweden

**Keywords:** Engineering, Materials science

## Abstract

Durability of cellulose-based insulation in power transformers is a key element to guarantee the reliability of power transmission systems. Under constant loading, continuous deformation is called creep, and its accurate prediction is essential for estimating the service life for constructions under dead load. Conducting decade-long experiments is technically possible yet practically unattainable at the design stage; therefore, we search for reliable methods that use shorter experiments (months) to predict long-term behavior (years). Extrapolation of measurement data often induces significant deviations from the actual response, even when the prediction time is only three to five times longer than the experimental data. Specifically, in this work, we study creep behavior, which is the dominating response in cellulose and related biopolymers for decade long static loading, especially in the presence of moisture. We analyze pressboards used in technical applications that exhibit a relaxation time of several hours, making the choice of an accurate material model challenging. By implementing three different approaches from the literature for modeling the creep behavior, we demonstrate a simple yet effective methodology to predict time spans longer than the experiment itself. The creep response of a precompressed pressboard has been analyzed, and with the presented data-driven method by means of an inverse analysis, a short-time experimental investigation is sufficient to characterize the creep properties, which then predicts the mechanical behavior for time spans five times longer than the experimental data’s duration. The study demonstrates this approach under $70\%$ RH (Relative Humidity) conditions, the same methodology may be used for different moisture levels in the future.

## Introduction

Experimental characterization is known to be valid within the time limit of the experimental data. For example, if we use an adequate material model and determine the material parameters by using a one-hour test, we may accurately predict response in any other configuration with another loading scenario but only up to one hour. It is unclear, in general, how we may use this test and determine a 5-hour response. The overall hypothesis is that a precise material model delivers precise prediction even outside of the experimental window. However, the model’s success is unknown until an experiment has been used for analysis. With this study, we aim at developing a methodology for estimating the predictive capacity of a given material model for longer duration than the short-term experimental campaign.

Creep behavior of polymers are studied in the literature^1,2^. In general, standard rheological models are used in order to incorporate viscous material response. Some modified rheological models are discussed for amorphous epoxy thermosetting polymers^3–5^ even for longer time durations^6^. Such models are often proposed and then validated by experiments only within the time limit of the underlying experiment. Yet there are no discussions about their accuracy in predicting longer time periods than tested. One well-established method for long-term prediction relies on accelerated experiments. For example in polymers, increased temperature accelerates the viscous deformation such that the short term measurement at a higher temperature may be used for a long-term prediction at a lower temperature^7–9^. This so-called time-temperature superposition principle employs a master creep curve from a collection of curves at different temperature values in order to determine a time-shift factor^10–12^ with a limited extrapolation in time^13^. The limitation is based on the fact that this accelerated test assumes that all material parameters depend on the temperature by the same manner, in other words, the underlying material is rheologically simple. In some cases, even for wood-based materials, this method has been used for predicting longer duration than in the experiments^14,15^.

Long term studies for materials under aging necessitates long term experimental campaign^16^ that poses a considerable hurdle for material development efforts. Accelerated tests^17^ are possible yet some aging related phenomenon’s rate is difficult to increase without altering underlying mechanisms^18,19^. More inventive experimental setups or inverse analysis methods are needed for a long-term prediction without using accelerated kinetics, herein, we follow the idea of testing the accuracy in extrapolation by varying the test duration^20^. Despite the fact that the method is empirical in nature, it may be useful for processes where an acceleration is challenging or not well understood, yet.

Paper-based materials consists of a combination of polymers such as cellulose, hemicellulose, and lignin. These polymers are the building blocks of fibers with very high aspect ratio, small diameter vs. large length. The structure of paper, created during paper production, generates a network of entangled fibers with irregular voids. Paper exhibits strongly orthotropic behavior that is the result of a fiber network with complex mechanical properties. The fiber network is inherently stronger and stiffer along the longitudinal axis of the fibers, whereas perpendicularly to the fibers, the network is much more pliable due to the high compressibility of the fibers and the availability of voids to accommodate the deformation of the fibers. Specifically in such cellulose based materials, aging is effected by the combined effect of temperature and moisture content^21^ as well as impacted by microfibril distribution^22^ that may be modeled with additional field variables^23^. The humidity effects are important to stress that an accelerated test is difficult to perform for such materials. Therefore, creep modeling is tedious owing to the complex and multiscale nature of wood^24^ and of engineering interest for paper based materials^25^. Several techniques do exist in the literature to model creep in cellulosic materials with remarkable difficulties^26^. In this study, we pursue a phenomenological approach to characterize the material response for a homogeneous medium under constant humidity to detect the predictive capacity of the proposed material model.

Many of solid insulation materials used in high-voltage equipment such as power transformers are fiber based. For example pressboard and paper are nowadays in use because of a favorable combination of dielectric and mechanical properties. Furthermore, most of paper-based insulation components are loaded in compression along the perpendicular direction to the fiber network, i.e. through the thickness of a paper sheet. The mechanical performance of the system relies on the stability of material. It is therefore relevant to investigate the long term-behavior of paper network under different environmental conditions. We propose a simple approach to test the accuracy in extrapolation. For pressboards, we apply a dead load and record the creep response in Sect. 2. Then by using a material model in Sect. 3, we determine the material parameters by means of a shorter duration of the data. With these parameters, we predict the material response for the complete duration in order to understand predictive capability of different material models in Sect. 4. The proposed methodology may be repeated for several temperature and humidity conditions analogously, which is left to future research.

## Experimental methods

From the variety of solid insulation materials used in power transformers, we use pressboards herein. This material undergoes several steps in manufacturing. It is mainly made from softwoods such as spruce and pine because of their long fibers (a matrix of lignin and hemicellulose) that leads to higher in-plane strength than papers made of shorter hardwood fibers. We refer to^27^ for a comparative analysis between spruce and other types. After the so-called Kraft cooking that fragments and dissolves lignin out of the cellulosic fibers, the so-called electrotechnical pulp is used to produce (cellulose) paper by refining (also called milling or beating) via passing through knives. This process leads to swelling, fibrillation, and shortening, which allows to control the end product’s mass density and air permeability, directly altering the mechanical properties. After the refining step, the pulp-water slurry reaches a board machine that lays wet sheets of different thicknesses. Around 30% of solid content is used in this step. A drying process is then applied where the final moisture content reaches a range of a few percent. Herein, we use a so-called precompressed pressboard (IEC 60641-3-1 type B.3.1. A) that is more suitable for applications where high mechanical and dielectric properties are required. We refer to^28^ for a more detailed explanation of the production methods of pressboards used in power transformers.

For the mechanical experiments, standard creep tests are conducted. A dead load is applied on the aforementioned pressboard where a predetermined mass is put on the specimen within a controlled chamber with a Relative Humidity, RH = $73\pm 1$% throughout the experiments. The mass applied on the specimen size results in a compressive stress 2.33 MPa that causes a creep response with a long relaxation time. Hence, several hours of data has been recorded. In total, 5 specimens were tested, yet caused by the spring back effect after manufacturing, only one test has been viable to use. Spring back is a common phenomenon related to the moisture uptake or discharge and change of dimensions^29^. We demonstrate a single experiment’s continuous data of 5 days. The deformation is collected by the Digital Image Correlation (DIC) method, which is an optical (non-contact) measurement by means of an image analysis^30^. The DIC implementation consists of three main steps: sample preparation, image recording, and image processing. If only one camera is used (2D DIC analysis), it is best suited for strain measurements in which there are modest deformations of planar surfaces. The sample surface needs to have a random texture that does not have a specific orientation and is non-periodic^31,32^. This pattern is achieved by (spray) painting, where by spraying a black color on a white background, random, black-and-white speckles are generated. Digital speckle photography is well suited for measurements on small objects^33^. For the recordings of the displacements, a digital camera and a constant (white) light source are needed.

The basic principle of DIC is to record a series of images of the sample during deformation, and then to calculate the degree of displacement and the strain field by tracking the pixels. For tracking the deformation, the Region Of Interest (ROI) is discretized in positions with a structured grid of stepsize in pixels. For each position, a circular area of a set radius in pixels is used to record the grayscale distribution. Hence, this area needs to be large enough to involve a unique speckle pattern. By selecting the radius too small, unrealistic noise is introduced, yet a subset radius parameter set too large will over-smoothen the results losing the precision. We have determined the ideal subset radius value based on identifying the smallest value that still prevents noisy strain data. The recommended procedure for this analysis is to drag the point to areas of high deformation to verify whether the curve fit remains accurate. We have tested along the horizontal line of the DIC result in the middle of the specimen by varying the subset radius—the middle is far away from boundaries and we expect to obtain a smooth strain value along this line. We have used Matlab code ncorr^34^ in this study. For all analyses, we use 10 pixel radius to generate the subsets with 1 pixel subset spacing to increase the resolution. The inverse method is iterative in a while loop with exit conditions: either the change between iterations becomes less than $10^{-6}$ (called diffnorm cutoff) or the number of iterations exceeds 50 iterations (called iteration cutoff). Each pixel is 0.056033 mm and we use 0.4729 as the correlation coefficient cutoff.

## Modeling approach

Creep response is caused by a viscous behavior of the underlying material. We may reason that the cellulose based materials are constructed by entangled fiber-like structures at different length-scales such that several relaxation times are acting simultaneously effected by this “fibrous” microstructure. We may interpret that two mechanisms play a role in the mechanical response: creep of the fiber material itself and creep related to the fibrous microstructure. Such “time-dependent” rupture mechanisms^35^ are investigated at the level of individual fibers^36^ as well as at the structural level as release of bonds between fibers and pull-out of fibers^37^. A multiscale method may be used to characterize the behavior by creating a digital twin of this microstructure with a suitable homogenization approach^38–40^. Yet the prediction accuracy of such an approach is linked to accurate modeling of the behavior and determination of material parameters at smaller length-scales. Instead, we follow a phenomenological approach based on proposing a function for the material’s response with the following restrictions:

- Firstly, the material model needs to be thermodynamically sound. This behavior means that the viscous behavior is dissipating energy. Based on rheological models, we generate material models by means of this restriction.
- Secondly, the model has to be valid in different loading scenarios. This functionality is guaranteed by using the tensor notation and normally not validated. There is a possible debate if the model reduction in rheology inhibits this property that we shortly discuss in the following.

In rheology, we use an analogy and relate force to stress and stretching to strain in order to introduce a one-dimensional model through linear springs and dashpots. We skip a detailed derivation of these models and refer to^41^ for models. The most general, linear, one-dimensional rheological model combines rates of stress with rates of strain

1 $$\begin{aligned} \begin{aligned} \sigma + q_1 \sigma ^{\bullet } + q_2 \sigma ^{\bullet \bullet } + q_3 \sigma ^{\bullet \bullet \bullet } + \dots = k_0 \varepsilon + k_1 \varepsilon ^{\bullet } + k_2 \varepsilon ^{\bullet \bullet } + \dots \ . \end{aligned}\end{aligned}$$

This linear relation is well established for hard materials such as plastics, alloys, and ceramics. For soft materials, gel type polymers, nonlinear material models are needed, their parameter determination is done elsewhere^42–44^. If a material response is modeled with first rate of stress and second rate of strain, this model reduces to the so-called 4-parameter solid model:

2 $$\begin{aligned} \begin{aligned} \sigma + q_1 \sigma ^{\bullet } = k_0 \varepsilon + k_1 \varepsilon ^{\bullet } + k_2 \varepsilon ^{\bullet \bullet } \ . \end{aligned}\end{aligned}$$

Experiments are conducted to determine the material parameters, $q_1$, $k_0$, $k_1$, $k_2$. In the isotropic case, this one-dimensional formulation is extended to a three-dimensional material equation by decomposing the stresses and strains into their deviatoric and spherical parts and utilizing the same or a different model to each of them separately. In the anisotropic case, we refer to^45–48^.

A well known experimental technique is a dead load experiment, where a uniaxial loading is instantaneously applied and held. In other words, $\sigma$ is given and constant in time. An often used solution of the aforementioned type of different equation, under constant stress, reads

3 $$\begin{aligned} \begin{aligned} \varepsilon = \frac{\sigma }{E_0} + \frac{\sigma }{E_1} \bigg ( 1 - \exp \Big ( -\frac{t}{\tau _1} \Big ) \bigg ) + \frac{\sigma }{E_2} \bigg ( 1 - \exp \Big ( -\frac{t}{\tau _2} \Big ) \bigg ) + \dots \ , \end{aligned}\end{aligned}$$

where $E_0$, $E_1$, $\dots$ and $\tau _1$, $\tau _2$, $\dots$ are seen as material parameters also given in form of spring and dashpot kind connections. The so-called creep compliance, $J = \varepsilon /\sigma$, is the response recorded, $\varepsilon$, per the given dead load, $\sigma$. This solution is only possible for a constant (in time) stress. The general linear relationship in Eq. (1) incorporates as many strain rates as necessary, where we have reduced the model to a 4-parameters model in Eq. (2) involving up to the second strain rate. The number of strain rates in the model determines the duration of deformation history acting to the stress. By increasing the number of rates, we incorporate a longer history of mechanical deformation. Consider a material with an unknown history dependence, we add *N*-many rates in order to allow the necessary history dependence, the creep compliance becomes

4 $$\begin{aligned} \begin{aligned} J = \frac{1}{E_0} + \sum _{\mu =1}^{N} \frac{1}{E_{\mu }} \bigg ( 1-\exp \Big (-\frac{t}{\tau _\mu } \Big ) \bigg ) \ . \end{aligned}\end{aligned}$$

This solution belongs to the generalized Kelvin (solid) model and the number of terms in the summation, *N*, is chosen on the needs in analysis. The parameters $E_0$ and $E_\mu$ are determined by setting the retardation times $\tau _\mu$ and using an inverse analysis based on an optimization algorithm that minimizes the squared error between the solution obtained from the parameters and the experimental results. Indeed, by increasing the number of terms in the summation, *N*, we expect to have better results. The first parameter, $E_0$, is an instantaneous (elastic) response. The set of $E_1$, $\tau _1$, $E_2$, $\tau _2, \dots$, $E_N$, $\tau _N$, are assumed to be independent and related to the viscous (creep) response.

One approach is to set *N* and then search for all creep parameters. In this approach, which we call a *viscous approach*, we simply define $E_0$, $E_1$, $\tau _1$, $E_2$, $\tau _2, \dots$, $E_N$, $\tau _N$ as unknowns such that $2N+1$ parameters need to be determined.

Another approach is to pre-select $\tau _1$, $\tau _2, \dots$, $\tau _N$, we call it a *logarithmic approach*. In the case of an experiment, say up to 1000 s, it is possible to use $\tau _\mu =\{10^{-1}, 1, 10, 10^{2}\}$ and then reduce the unknowns to $N = 5$ including $E_0$.

For creep tests with long loading times, a large number of elements in the chain is needed which would make the inverse identification procedure ill-posed and predictions error-prone. Instead of considering the many moduli in the chain as independent variables, another approach is based on a continuous (fit) function by using a so-called retardation spectrum. The well-known *retardation spectrum*^49–52^ is helpful to increase the quality of the fit and improve the computational efficiency. For this sense, we employ the following form

5 $$\begin{aligned} \begin{aligned} \frac{1}{E_{\mu }} = L(\tau _\mu ) \ , \quad L(\tau ) = a \tau ^b \ , \end{aligned}\end{aligned}$$

in which $L(\tau )$ is the continuous retardation spectrum with a possible power law empirical form described by two terms, *a* and *b*, to be fitted irrespective of *N*. This approach works for many materials where there is one dominating region for retardation time. For a composite with a mixture exhibiting several retardation times, the chosen form of $L(\tau )$ may be chosen differently. The choice is a phenomenological approach without restrictions than the simplest as possible that is capturing the material behavior accurately.

We apply all of these approaches in the following in order to test their success in predicting the material response beyond the experimental duration. In summary, we use three approaches:

- *Viscous approach*: Moduli and time constants are determined as unknowns, to-be-determined parameters read $E_0$, $E_1$, $\tau _1$, $E_2$, $\tau _2$, ...
- *Logarithmic approach*: In order to determine compliance values, $1/E_1$, $1/E_2$, ...$1/E_7$, a predefined (in log base) retardation time list is used, $\tau _\mu =\{10^{-1}, 1, 10, 10^{2}, 10^{3}, 10^{4}, 10^{5}\}$.
- *Spectrum approach*: Finding *a* and *b* in $L(\tau ) = a \tau ^b$ allows to determine compliance values in the same predetermined retardation time list.

All approaches are valid but depend on different assumptions. For a fair comparison, we select the time list the same for the *logarithmic approach* and *spectrum approach*. After inspecting the whole spectrum, we obtain the minimum number of unknowns in the *viscous approach*. Once parameters are determined, the instantaneous response and the retardation spectrum are known. In the simplest case, with a co-linearity assumption, the 4-parameter solid material model reads

6 $$\begin{aligned} \begin{aligned} \sigma _{ij} + q_1 {\sigma _{ij}^{\bullet }} = k_0 \varepsilon _{ij} + k_1 {\varepsilon _{ij}^{\bullet }} + k_2 {\varepsilon _{ij}^{\bullet \bullet }} \ . \end{aligned}\end{aligned}$$

We refer to (Table 1.2^41^) for a discussion about the assumption of independent material properties as given in Eq. (3) and use the relation between Eq. (4) for obtaining a direct relation, as follows:

7 $$\begin{aligned} \begin{aligned}&\tau _1 = \frac{1}{\lambda _1} \ , \quad \tau _2 = \frac{1}{\lambda _2} \ , \quad \\&\quad k_0 - k_1 \lambda _1 + k_2 \lambda _1^2 = 0 \ , \quad \frac{1}{E_2} = \frac{1-q_1\lambda _2}{k_2 \lambda _2 ( \lambda _1-\lambda _2 ) } \ , \quad \frac{1}{E_1} = \frac{1-q_1\lambda _1}{k_2 \lambda _1 ( \lambda _2-\lambda _1 ) } \ , \quad E_0 = k_0 \ . \end{aligned}\end{aligned}$$

With these four equations, the material parameters are determined from measurable coefficients and the material model is implemented in a finite element method in order to simulate different loading conditions for any given geometry, for such engineering examples, we refer to^53^. We emphasize that the material parameters are obtained by an inverse method that has been undertaken by minimizing the objective function that is the sum of squared error between measured strain and estimated strain. We use a linear loss from SciPy^54^ optimization toolkit the Levenberg–Marquardt algorithm. This method begins from an initial guess of parameters and finds the parameters minimizing the cost function that is depending on this initial guess. Since the nonlinear regression problem may have several solutions, the initial guess is rather important to obtain the global minimum and we enforce this by taking 100 randomized initial guess values (sometimes called a Monte–Carlo approach). Within the solution of all minimum values, the global minimum is declared as the “best” parameters that are employed for plotting. The initial guess values are realistic values for moduli between $10^6$ and $10^8$; for time constants between $10^{-3}$ and $10^8$. This optimization procedure is solved in Python by using open-source packages, the generated code is explainable and open for further use and development for other materials.

## Results and discussion

**Fig. 1:**
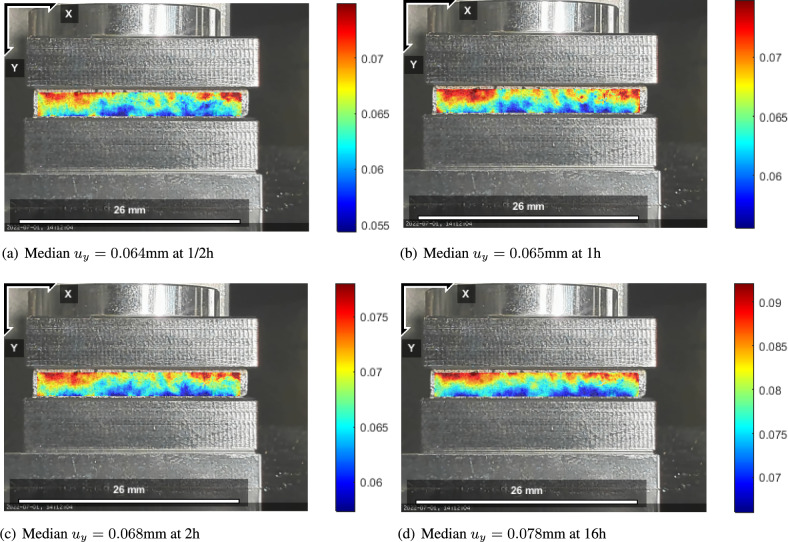
The vertical displacement field of the specimen: (**a**) $u_{y}$ at 1/2h; (**b**) $u_{y}$ at 1h; (**c**) $u_{y}$ at 2h; (**d**) $u_{y}$ at 16h, deviation from a linear distribution is visible in the displacement due to the heterogeneity within this fibrous material.

A dead load is applied and held constant for 5 days by taking a photo every 30 min. The total 120 h of measurement time is $4.32\times 10^5$ s used in plotting. Digital Image Correlation (DIC) results of the displacement field are seen in Fig. 1 for different time instants.

**Fig. 2:**
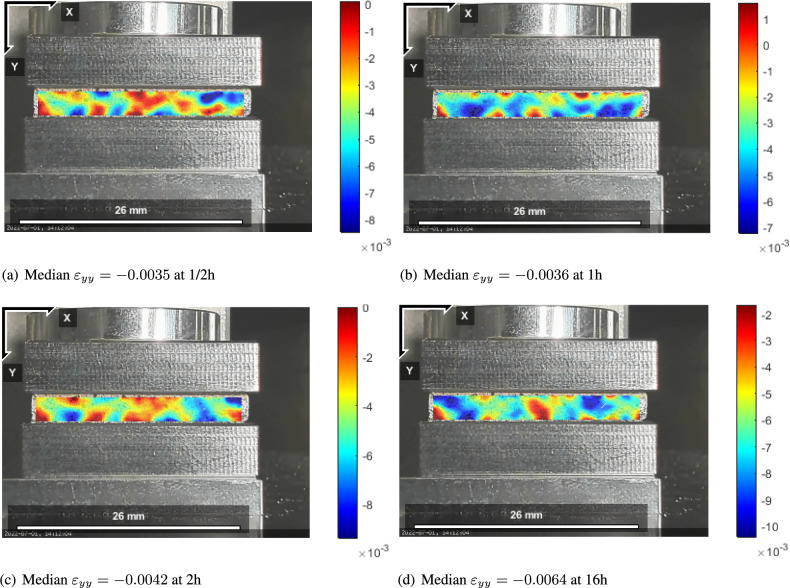
Normal strain field of the specimen: (**a**) $\varepsilon _{yy}$ at 1/2h; (**b**) $\varepsilon _{yy}$ at 1h; (**c**) $\varepsilon _{yy}$ at 2h; (**d**) $\varepsilon _{yy}$ at 16h, as the strain field is obtained by a space derivative from the heterogeneous displacement distribution, strain is non-uniform as well.

Each displacement field is obtained by the given length per pixel information. Obviously there are edge effects building a boundary layer in a similar manner in all times. We emphasize that the recorded displacement field shows a non-uniform distribution, which arises from the material heterogeneity—pressboard is of a fibrous microstructure with dispersive bundles of fibres. Despite this non-homogeneity, the homogenized models are useful as demonstrated in the following. Through thickness, along the *y*-axis, the deformation is maximum on top and zero on the bottom that is clamped. A monotonous increase of the median is visible, where the median is simply the arithmetic mean of the data. By taking the numerical derivative along the thickness, we obtain normal strain within the Region Of Interest (ROI) as shown in Fig. 2.

We stress that the calculation of the mean value has more accuracy for each time instant with respect to the measurement of platen positions (stroke). In this way, boundary effects are visible and potential adherence effects may be detected. Too high friction on the plate would lead to a barreling phenomenon^55^. Non-uniform strain distribution has been recorded in the literature for such fibrous materials^56^, where large strain variations are condoned by the large local variations in mass density. We propose to expect the same variation in the stress field as well so that the same material model holds locally. In other words, we observe the same variation within the ROI captured in DIC as seen in Fig. 2. Therefore, we conclude that the irregularities are caused by the material distribution and not experimental inconsistencies. By taking the mean value (median), we expect to see a monotonous change in the response that is then used for parameter fitting. In this material with a particularly long retardation time, loading speed is adequate to assume instantaneous. The constant (compressive) stress of 2.33 MPa is utilized in order to determine the initial modulus, $E_0$, by means of the measured strain initially. We use a linear material model for the elastic response. This simplification introduces a negligible error since the strain in the elastic regime is small with respect to the creep response. For fitting the strain data, we use this stress value in the inverse analysis.

**Fig. 3:**
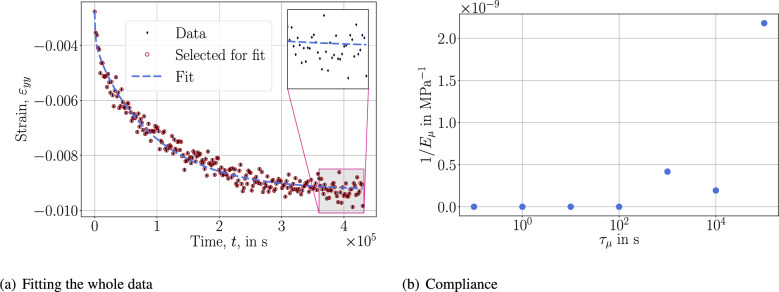
Fitting with the *logarithmic approach* and $N=7$ with the shown retardation spectrum.

By using the whole 5-day (120 h) data, we obtain a fit to the response as demonstrated in Fig. 3. This result is obtained by using the *logarithmic approach* that is more or less the standard approach in creep tests and their inverse analyses.

**Fig. 4:**
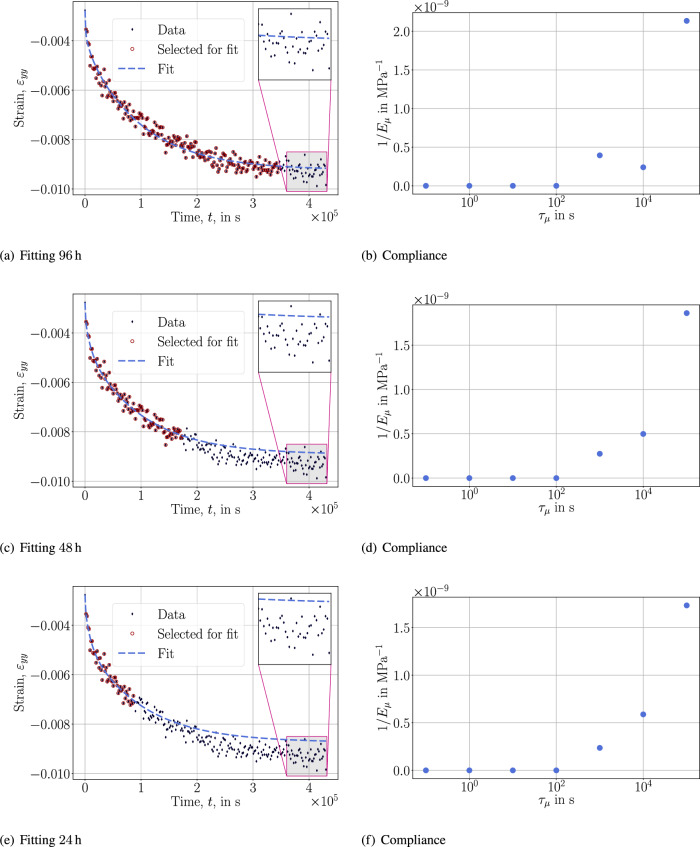
Fitting and compliance plots with the *logarithmic approach*.

Because the duration is up to 5 days, we use $\tau _\mu =\{10^{-1}, 1, 10, 10^{2}, 10^{3}, 10^{4}, 10^{5}\}$ second in order to cover the whole time interval. Spectrum of compliance reveals that 2 distinct retardation times, namely $10^3$ s and $10^5$ s, are dominating. In other words, there are different mechanisms affecting the creep response, the underlying material needs at least 2 viscous parameters in order to involve both behaviors. Here, we have used 7 retardation times because of the logarithmic approach for representing 2 dominating viscous phenomena. The fit is accurate, as expected, demonstrating the success of the *logarithmic approach* within the experimental duration. We test its fidelity by reducing the data used for fitting yet estimate 5 days to measure its predictive capability outside of the experimental duration in Fig. 4.

**Fig. 5:**
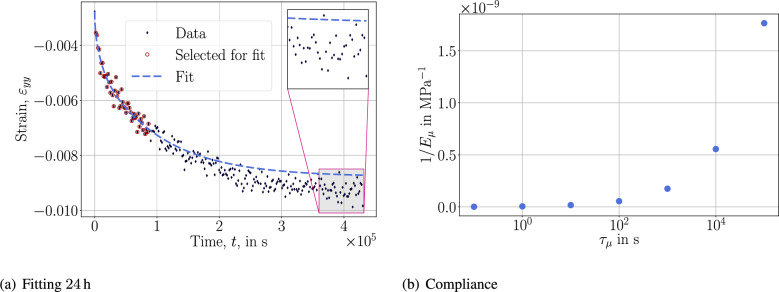
Fitting and retardation spectrum with the *spectrum approach*.

The test with 96 h (4 days) estimates the compliance well such that the prediction is accurate (within the bounds of the data). However, once the selected duration is shortened to 2 days, the order of dominance in $\tau _\mu$ changes, resulting an undershooting in the prediction. Simply stated, despite the material model’s success in modeling the response within the time interval of the data, it lacks the capacity to extrapolate with the same accuracy. This approach uses 7 unknowns to determine the model. The same inadequacy to extrapolate is observed in the *spectrum approach* that uses only 2 unknowns, where we stress that the same $\tau _\mu$ is used to determine basically the same fit. In this approach, the power law spectrum fails to model more than one dominance in the compliance spectrum. More general forms may be used but they are not expected to amend the lack of extrapolation capacity of the *logarithmic approach* as visible in Fig. 5.

**Fig. 6:**
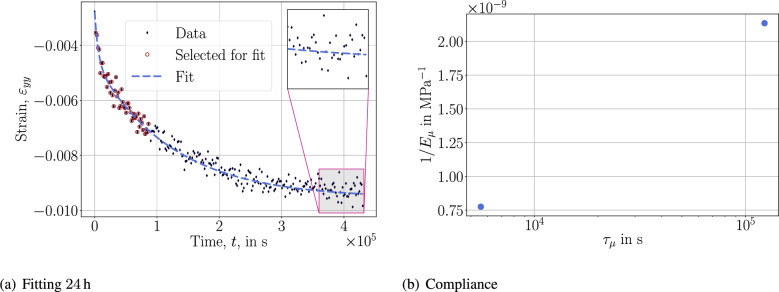
Fitting and compliance plots with the *viscous approach*.

As we have observed that the material possesses 2 characteristic time constants, we model the *viscous approach* with $\tau _1$ and $\tau _2$ as unknowns. In this way, 4 unknowns are determined in the inverse analysis, $E_1$, $E_2$, $\tau _1$, and $\tau _2$. The extrapolation capacity of this model is accurate as demonstrated in Fig. 6. The scatter is less than $0.1\%$ in strain values that is adequate for industrial use. We compile the parameters for the successful *viscous approach* in Table 1.

**Table 1:** Results of retardation spectrum for reduced data fitting, only 1-day data is used for predicting a 5-day test, the parameters from the fit are to-be-used in Eqs. (6) and (7).

Parameter	Value	Unit
Modulus, $E_0$	838	MPa
Modulus, $E_1$	469	MPa
Retardation time, $\tau _1$	$123.2 \times 10^{3}$	s
Modulus, $E_2$	1291	MPa
Retardation time, $\tau _2$	$5.6 \times 10^{3}$	s

We accentuate that the main goal is to predict accurate compliance values for set or sought after retardation times. The typical increase in compliance over time demonstrates that creep is reaching a value. Duration of attaining this state is given by the moduli per time constant (characteristic time describing the lag of the response) such that for this material using time constant as unknowns seems to be a good option in general.

As demonstrated, 1 day of testing is adequately delivering an accurate prediction up to 5 days. In this manner, we have developed a simple procedure to estimate the accuracy of the fit by extrapolation from 24 h to 120 h. With cellulose fiber material as an example, we have used this procedure to predict long-term creep behavior based on short-term creep data. The approach also examines how short the characterization testing time may be selected in order to accurately predict long-term deformation. In this setup, less than 1-day test data has been found insufficient. Despite using a heterogeneous fiber material as an example with notable non-uniform strain distribution, as short times as 1/5 of the prediction time prove sufficient with an error less than the measurement scatter. We fail to claim that the method may be scaled in time that 1 year test may predict 5 year performance. Indeed the general vision is to understand possibilities for using material models longer than the test, at least, we have demonstrated its success in terms of days. Longer experimental campaigns may shed light upon further insight of the model’s capabilities.

## Conclusion

Material modeling may be conducted by a phenomenological approach based on experimental results that has the weakness of accuracy within given bounds of experimental setup. If a short term mechanical response is recorded and used in a material model, there is no guarantee that the same accuracy is reached for a long term response. Therefore, we propose a straightforward method to test for a particular material about the extrapolation characteristics of a creep model from the literature. For a cellulose based material, we have demonstrated the capacity of such an extrapolation depending on the chosen shorter term dataset. Despite the approach being simple, it is key to understand that a selection procedure of shorter term duration is only possible experimentally. We have observed that

- By using moduli and characteristic times as unknowns, the obtained results are encouraging.
- Inverse analysis is robust by using several initial values.
- A material model is employed that is a differential relation without explicit time dependence, thus, it is suitable to be used in numerical simulations.
- The obtained ratio of 1 to 5 demonstrates that 1-day-test data has been adequate to predict 5 days precisely.

This method requires constant temperature and humidity values during the dead-load-creep experiment. By testing for several values, if the material model is still valid, it may be possible to cover a range of realistic conditions. Herein we assume that the dependence on temperature and humidity may be of different type such that an acceleration is not possible by using a master curve approach. This distinction is needed for complex materials. Limitations of this method needs to be further investigated by longer tests in order to see if the same ratio may be applicable in such cases. Additional possible investigations are about the surface characteristics and their role on the overall behavior due to the plastification of the surface leading to flattening at a different time scale.

## Data Availability

Data sets generated during the current study are available from the corresponding author on reasonable request.
